# Non-Invasive Acoustical sensing of Drug-Induced Effects on the Contractile Machinery of Human Cardiomyocyte Clusters

**DOI:** 10.1371/journal.pone.0125540

**Published:** 2015-05-11

**Authors:** Angelika Kunze, Daniella Steel, Kerstin Dahlenborg, Peter Sartipy, Sofia Svedhem

**Affiliations:** 1 Department of Applied Physics, Chalmers University of Technology, Göteborg, Sweden; 2 Cellectis AB, Göteborg, Sweden; 3 Systems Biology Research Center, School of Bioscience, University of Skövde, Skövde, Sweden; Mayo Clinic, UNITED STATES

## Abstract

There is an urgent need for improved models for cardiotoxicity testing. Here we propose acoustic sensing applied to beating human cardiomyocyte clusters for non-invasive, surrogate measuring of the QT interval and other characteristics of the contractile machinery. In experiments with the acoustic method quartz crystal microbalance with dissipation monitoring (QCM-D), the shape of the recorded signals was very similar to the extracellular field potential detected in electrochemical experiments, and the expected changes of the QT interval in response to addition of conventional drugs (E-4031 or nifedipine) were observed. Additionally, changes in the dissipation signal upon addition of cytochalasin D were in good agreement with the known, corresponding shortening of the contraction-relaxation time. These findings suggest that QCM-D has great potential as a tool for cardiotoxicological screening, where effects of compounds on the cardiomyocyte contractile machinery can be detected independently of whether the extracellular field potential is altered or not.

## Introduction

Heart failure and arrhythmias are the major causes of morbidity and mortality in many countries. Besides genetic and life style reasons, many adverse cardiac events are induced by detrimental off-target effects of non-cardiac drugs [1]. To reduce the number of drug-induced arrhythmias and other functional deficiencies, there is an increasing demand for further development of cardiotoxicity assays using human material for evaluating existing and new chemical entities in pre-clinical studies.

In cardiotoxicology research, cell models used for drug screening should be species relevant, provide high reproducibility, and exhibit specific markers and functional similarities to adult human cardiac myocytes. All of these features have been demonstrated for cardiomyocytes derived from human pluripotent stem cells (hPS) [2–4], including diseased phenotypes [5]. Specifically, spontaneously beating hPS-derived cardiomyocytes in a cluster format (CMC) can be obtained in large volumes and have become interesting for toxicology research [6,7]. These cell clusters range in size from 200–300 μm in diameter and exhibit specific markers and functional similarities to adult human cardiac myocytes [8]. They are considered to be an excellent *in vitro* tool for studies of human cardiomyocyte function and are applied for pre-clinical cardiac safety pharmacology assays [9–15].

The combination of appropriate cells and suitable assay formats is a key to successful drug discovery, as well as to increase the fundamental understanding of cell properties. With respect to assay development, there is a need for real-time, label-free monitoring (also referred to as sensing) of rare cell function using array formats. Towards this end, advances have been made with respect to detection of changes in optical or electrochemical properties of cells [15–17], whereas techniques directly measuring changes in the mechanical properties of cells in vitro are largely lacking. The tight link between mechanical properties of cells and important cell processes (e.g. chronotropic events) suggests that acoustic methods (probing viscoelastic properties) have potential, alone or in combination with other techniques, in cell-based drug screening platforms [18,19]. One acoustic technique, the quartz crystal microbalance (QCM) technique, has been successfully applied to studies of attachment and subsequent spreading of cells at the surface of the QCM sensor [20,21], changes in cells exposed to cytomorphic agents [21–23], exocytotic events in neural cells on the sensor surface [24], pigment redistribution in melanophores [25], as well as activation of surface-confined platelets [26]. Furthermore, QCM has been applied to detect beating of cardiomyocytes, grown in a monolayer on the sensor surface [27], and to detect spontaneous beating of hPS-CMCs [28]. These findings show the potential of the QCM technology as a platform for monitoring of CMCs non-invasively, in a label free and real-time manner, aiming not only for the detection of chronotropic characteristics such as, e.g., arrhythmias, but also for properties of the cardiomyocyte contractile machinery, including changes of the QT interval (the time from the beginning of the Q-wave to the end of the T-wave in the electrical cycle of the heart, i.e., the time between the electrical depolarization and repolarization of the ventricles). Changes in the QT interval are a well-established marker for ventricular tachyarrhythmias, e.g., torsades de pointes with a risk for sudden death [1].

**Fig 1:**
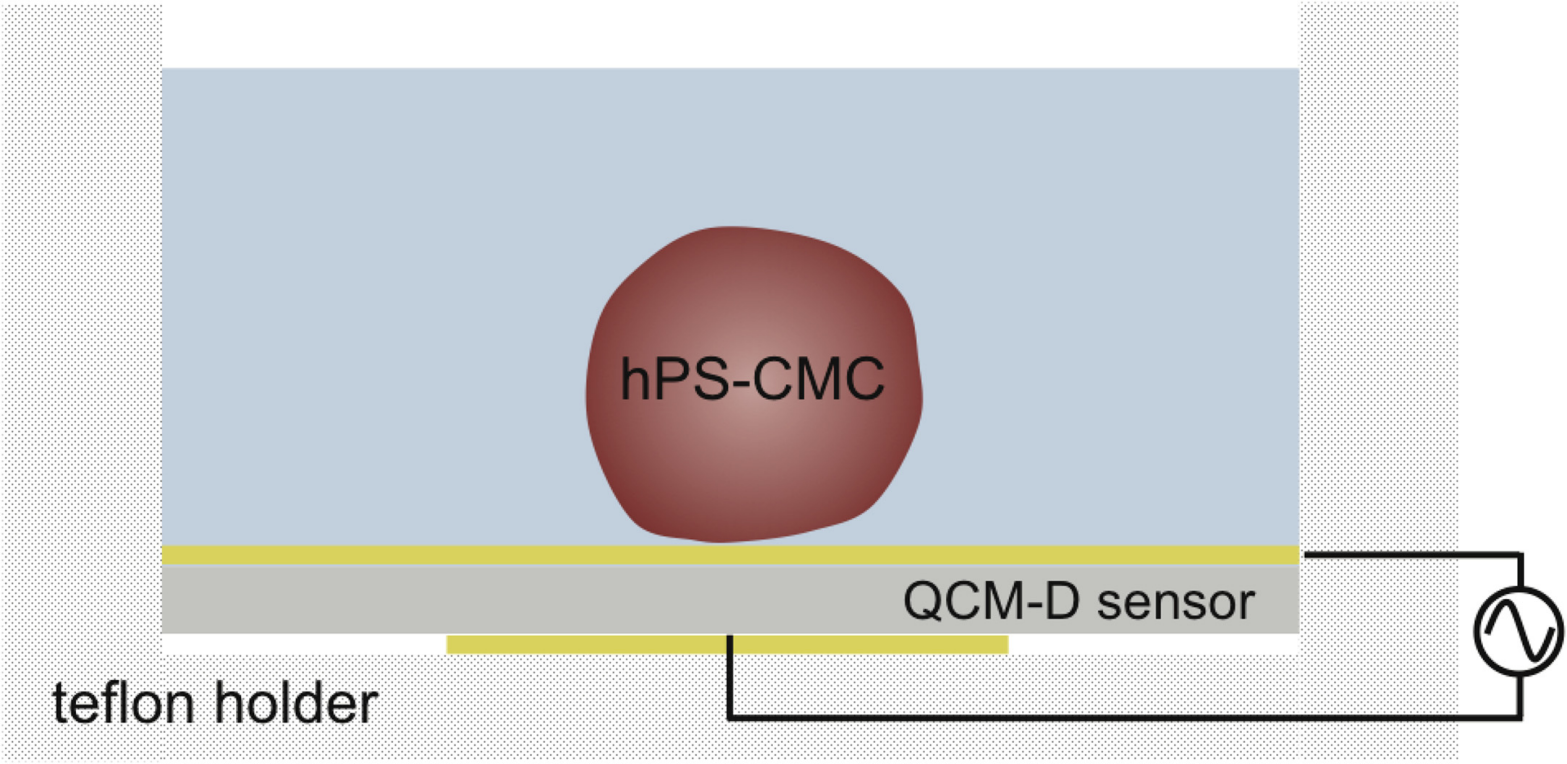
Schematic drawing of the experimental set-up.

This study addresses the monitoring of mechanical (viscoelastic) properties of individual cell clusters by acoustic sensing using QCM with dissipation monitoring (QCM-D). An open QCM-D module was used for the detection of effects induced by well-known model compounds on the spontaneous beating of hPS-CMCs (see Fig 1). Chronotropic effects were probed by addition of isoproterenol or high doses of E-4031. Low doses of E-4031 or nifedepine, both well-established model drugs to probe the electrochemical cycle of CMCs, were added to examine changes in the QT interval. In addition, we also incubated the cells with cytochalasin D, a potent inhibitor of actin polymerization, to independently probe for changes of the contraction-relaxation-cycle of the CMCs.

Based on these results, we suggest QCM-D, alone or in combination with another technique (e.g. microscopy or impedance spectroscopy), to be an attractive alternative to existing cardiotoxicity screening platforms.

## Materials and Methods

Unless otherwise stated chemicals were from commercial suppliers and used as received. Water was purified (filtered and deionized until a resistivity of 18 MΩ cm) using a MilliQ unit (Millipore, France). Nifedipine, isoproterenol (the bitartrate salt), E-4031, and cytochalasin D were purchased from Sigma.

Surface preparation steps of polystyrene-coated QCM-D sensors (Q-Sense AB, Sweden) were as follows. QCM-D sensors were cleaned by a UV-O_3_ treatment for 5 min followed by sterilization in 70% ethanol and rinsing with sterile water.

hPS-CMCs derived from the human embryonic stem cell line SA121 and showing molecular markers typical for cardiac cells were obtained from Cellectis AB (Göteborg, Sweden). Briefly, CMCs were maintained in knockout Dulbecco’s Modified Eagle Medium (DMEM) medium supplemented with 20% fetal bovine serum (FBS), 1mM GlutaMAX, 0.1 mM β-mercaptoethanol, 1% Minimal Essential medium (MEM) non-essential amino acids, and 1% penicillin as described previously (all components purchased from Invitrogen) [8]. For QCM-D experiments, the sensors were placed in the central well of a humidified IVF culture dish, to keep the underside as clean and dry as possible during the cell attachment phase. Single clusters were positioned centrally on fibronectin coated sensors in a droplet of medium and incubated without further interference for 4–5 days in standard cell culture conditions (5% CO_2_ and 95% humidity).

The QCM-D experiments were performed using a Q-Sense instrument of the E-Series combined with the open module QOM 401 (for details we refer to the supplier) and QCM-D sensors with a fundamental frequency, *f*_*0*_, of 5 MHz (Q-Sense AB, Sweden). The sensors onto which clusters had been grown (see above) were carefully removed from the wells of the culture dishes and the liquid underneath the sensor (and the lower electrode) was gently blotted and dried with tissue. Care was taken not to perturb the cluster attached to the upper electrode during the sensor mounting steps. The experimental set-up is schematically depicted in Fig 1. All experiments were performed at 37°C, and CMCs were immersed in liquid carefully added on top. Spontaneous beating of hPS-CMCs immersed in medium was monitored by QCM-D for 20 min. The model compounds (and diluent controls water and DMSO) were added by carefully pipetting 1 μl of stock solution to medium covering the CMCs. Measurements were recorded at the fundamental frequency with the highest sampling rate in order to ensure detection of rhythmic cell. Cluster beating (and continued attachment) was verified periodically throughout the QCM-D measurement. Mean values and sample standard deviations were calculated based on individual experiments given in the ESI. Experiments were repeated 3 to 4 times to ensure reproducibility.

## Results and Discussion

Experiments were performed to demonstrate the potential of QCM-D for detection of drug-induced chronotropic effects in beating CMCs, as well as effects on the properties of the contraction-relaxation cycle, in an attempt to monitor signals that may represent changes in the action potential duration and the QT interval.

### Chronotropic effects induced by isoproterenol or high doses of E-4031

Two model drugs (isoproterenol and E-4031), with well-established and different effects on cardiac activity, were added, in separate experiments, to spontaneously beating CMCs to probe for chronotropic effects. Isoproterenol is a non-selective beta-adrenergic agonist, similar to adrenaline, and is known to increase the beating rate of stem cell-derived cardiomyocytes [29–31] and hPS CMCs [14]. E-4031 is a specific hERG channel blocker, which has been shown to prolong the QT-interval and to provoke arrhythmia if added at high concentration (> 100 nM) to stem cell-derived cardiomyocytes [29] or hPS CMCs [2,15,32].

**Fig 2:**
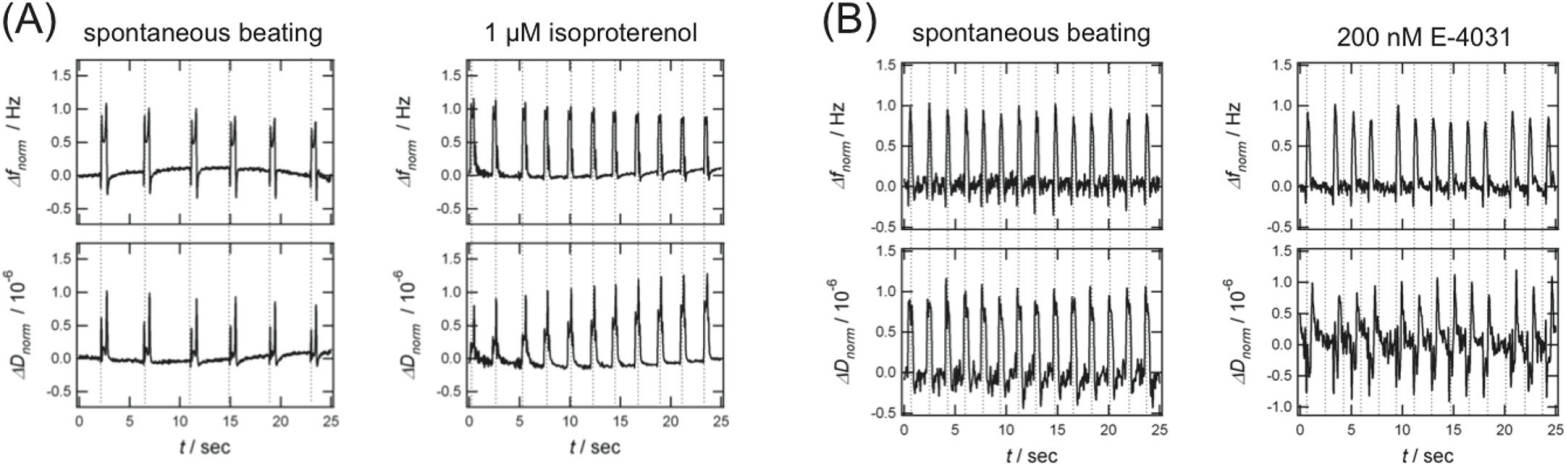
Detection of chronotropic effects. Frequency (top) and dissipation (bottom) time curves obtained with a spontaneously beating hPS-CMC (left panel, A and left panel, B), before and after addition of 1 μM isoproterenol (right panel, A) and 200 nM E-4031 (right panel, B).

Before addition of isoproterenol, we observed spontaneous beating of the cardiomyocyte cluster with a stable beating rate of 14–15 bpm (Fig 2A, left panel). After addition of isoproterenol to a final concentration of 1 μM, an immediate increase of the beating rate to 25–27 bpm was observed (Fig 2A, right panel). A different behavior is illustrated in Fig 2B, where a high dose of E-4031 (final concentration of 200 nM) was added to the CMC. By comparing the QCM-D signal of the spontaneous beating of a cardiomyocyte cluster (Fig 2B, left panel) with the beating of the same cluster after addition of 200 nM E-4031 (Fig 2B, right panel), we clearly observe an arrhythmic beating pattern of the CMC with QCM-D.

**Fig 3:**
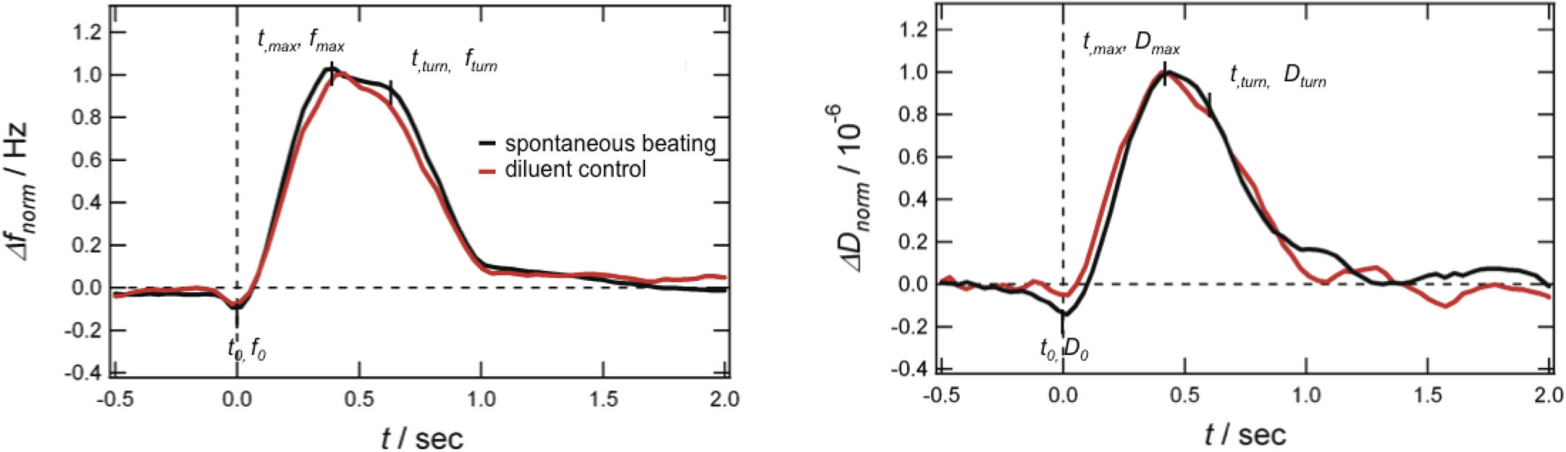
Characterization of a single beat. Frequency (left) and dissipation (right) time curves obtained with a spontaneous beating hPS-CMC (black), before and after addition of diluent (water) control (red). Frequency and dissipation shifts are normalized by setting maxima values to 1. The first minima is set to time *t*_*0*_ = 0 sec.

An important objective of the present work was to investigate whether QCM-D can be used for the characterization of a single contraction-relaxation cycle of a CMC. For this purpose, the beating cycle was studied in more detail to allow for the detection of changes in the duration of a single contraction-relaxation cycle. Fig 3 shows the QCM-D *∆f* and *∆D* versus time curves of a single beating cycle of a spontaneously beating CMC attached to the QCM-D sensor surface. The frequency and dissipation shifts have been normalized to facilitate the comparison between different CMCs (we observed the amplitude of the QCM-D signal to vary for different CMCs, and also during a single experiment, presumably due to evaporation of liquid in the open QCM-D module). To help comparing single contraction cycles of the same cluster before and after addition of compounds, the recorded data were normalized by setting the first minimum of the signal to time *t* = 0 sec and setting the amplitude of the first maximum in each of the QCM-D signals to 1 (i.e., at times *t = t*_*max*,*f*_ and *t = t*_*max*,*D*_ for the frequency and the dissipation shift, respectively, as indicated in Fig 3). After the first maximum at *t*_*max*_, a decrease of the signals to the baseline is observed, where in most of the experiments, a slow decrease is followed by a fast decrease at a turning point defined as *t = t*_*turn*,*f*_ and *t = t*_*turn*,*D*_. It is interesting to note that the shape of the recorded QCM-D signal is very similar to the cyclic waveform of the electrochemical signal (i.e., extracellular field potential) of a microelectrode array [33]. However, we can largely exclude that QCM-D detects changes in ion concentration or the membrane potential as such since the changes in ionic strength of the media due to Ca^2+^-fluxes of the beating CMC are small and the sensor surface is grounded [28]. In a previous study, the origin of the observed QCM-D signals from cells adhered to the sensor surface where they were undergoing morphological changes was discussed [21]. In brief, it was suggested that the QCM-D signals are mainly due to secondary, viscoelastic effects in the layer closest to the sensor surface. One may also consider the QCM-D signal to depend on shape deformation of the hPS-CMC caused by changes of the shear modules or the bending rigidity of the cell cluster [34]. Using giant unilamelar liposomes as cell mimics, it has been shown that electrically induced shape deformation led to a frequency shift of the QCM signal, related to the bending rigidity of the liposomes [35].

### Effects induced by E-4031 or nifedipine, compounds with known impacts on the CMC electrochemical cycle

**Fig 4:**
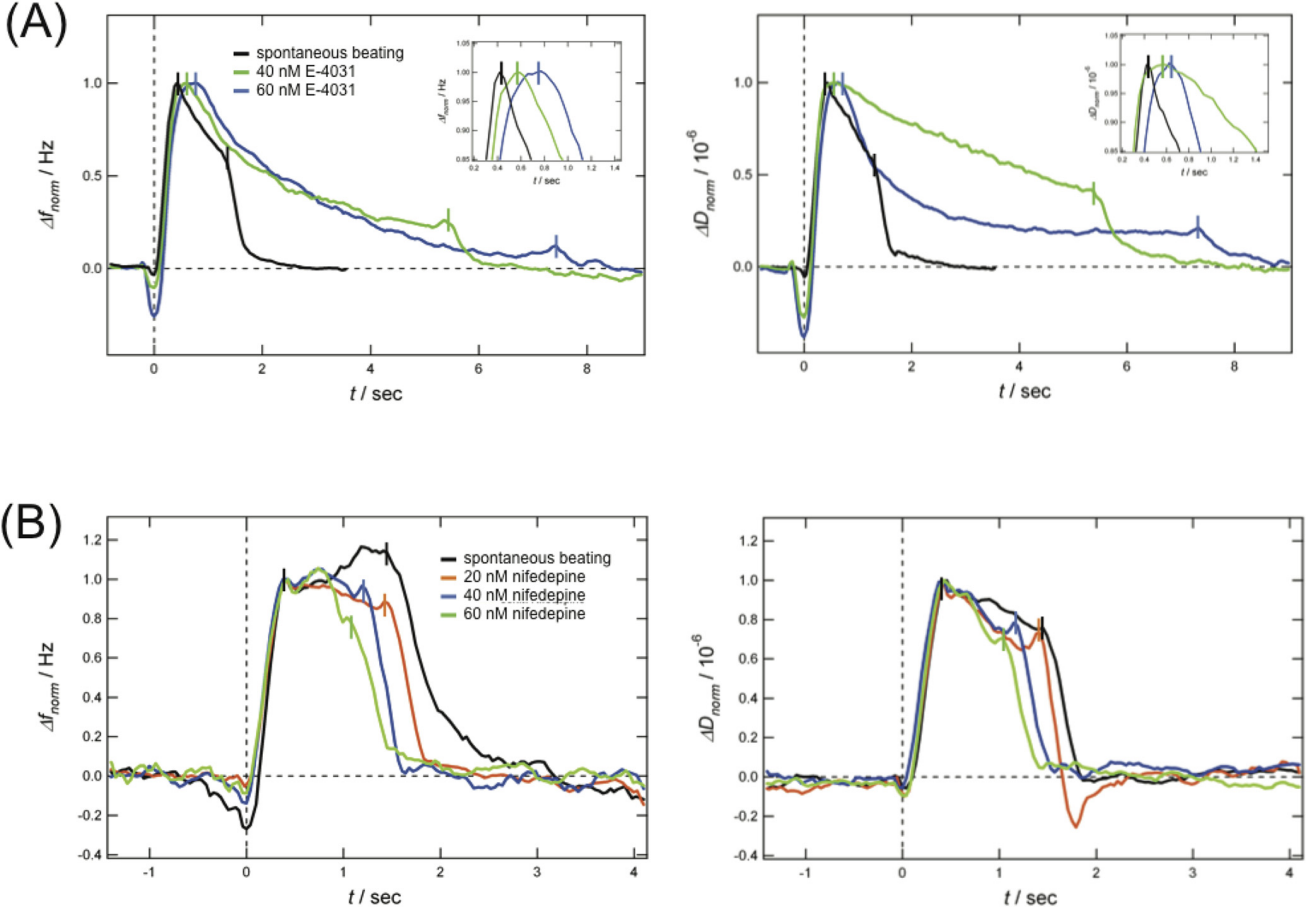
Detection of drug-induced prolongation and shortening of the QT interval. Frequency (left) and dissipation (right) time curves obtained with a spontaneous beating hPS-CMC (black), before and after stepwise addition of (A) E-4031 (green, 40 nM and blue 60 nM) and (B) nifedepine (red, 20 nM, blue, 40 nM and green, 60 nM).

Fig 4A shows how the normalized QCM-D signal of a single beating cycle of a beating CMC changes after stepwise addition (40 nM and 60 nM) of the hERG channel blocker E-4031. We can clearly observe a shift of *t*_*max*_ and *t*_*turn*_ after addition to longer times compared to *t*_*max*_ and *t*_*turn*_ of the spontaneously beating CMC. Changes of *t*_*max*_ and *t*_*turn*_ relative to the values of the spontaneously beating CMC are summarized in Table 1. The increase of both *t*_*max*_ and *t*_*turn*_ can be interpreted as an increase in the action potential duration typically observed in hPS CMCs treated with E-4031 [2,15], and may thus be used as a surrogate measure of the QT-interval.

**Table 1:** Changes in the contraction cycle of beating CMCs monitored by QCM-D upon the addition of E-4031 and nifedepine.^a^

		*∆t*_*max*_	*∆t*_*turn*_	
E-4031	40 nM	0.3 ± 0.2	3.0 ± 0.4	n = 3
	60 nM	0.7 ± 0.2	4.6 ± 0.3	n = 3
Nifedepine	20 nM	-	-0.15 ± 0.02	n = 3
	40 nM	-	-0.24 ± 0.05	n = 3
	60 nM	-	-0.45 ± 0.13	n = 3

^a^Relative changes *∆t*_*max*_ and *∆t*_*turn*_ are calculated as follows: $\Delta t_{max}=\frac{t_{max,drug}-t_{max,spontan}}{t_{max,spontan}}$ and $\Delta t_{turn}=\frac{t_{turn,drug}-t_{turn,spontan}}{t_{turn,spontan}}$.

Similarly, in Fig 4B the effect of stepwise addition of nifedepine (20 nM, 40 nM, 60 nM) to a spontaneous beating CMC is shown (values of ∆*t*_*turn*_ are given in Table 1). In this case, a reduction in *t*_*turn*_ is observed upon addition of the dihydropyridine calcium channel blocker that primarily blocks L-type calcium channels whilst *t*_*max*_ remains unaffected. Since nifedepine is known to shorten the QT-interval[14,36] we conclude *t*_*turn*_ to the characteristic of the QCM-D signal related to the QT-interval.

### Effects induced by cytochalasin D, known to interfere with the cytoskeleton

In order to verify that the observed QCM-D signals are related to the contraction of the hPS-CMC, we added cytochalasin D, a potent inhibitor of polymerization of actin, an essential component of the cytoskeleton. In cardiomyocytes, the cytoskeleton is a critical junction for mechanotransduction. It provides an intracellular structure for transmitting contractive forces. In studies using single cardiomyocytes, it has been shown that the contractility of rat ventricular myocytes is significantly depressed by short-time (minutes) exposure to cytochalasin D (40 μM) whilst only small changes of the intracellular calcium ([Ca^2+^]_i_) transient were observed [37].

**Fig 5:**
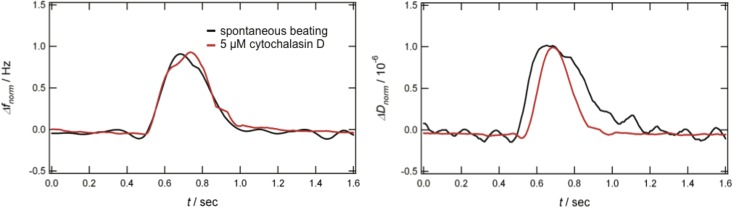
Effect of cytochalasin D. Representative frequency (left) and dissipation (right) time curves obtained with a spontaneous beating hPS-CMC (black), before and after addition of 5 μM cytochalasin D (red). The full width at half maximum of the dissipation signal decreases about 50% ± 14% (calculated by a set of three experiments). The value of the full width at half maximum is given by the difference between the two times at which the frequency and dissipation shifts, respectively, is equal to half of its maximum value.

Here, spontaneously beating hPS-CMCs were exposed to 5 μM cytochalasin D to probe for changes in the contraction-relaxation cycle. Fig 5 shows the single beating profile of the cluster before (spontaneously beating CMC, black curve) and after addition of cytochalasin D (red curve). The shape of the frequency signal remains essentially the same, whereas the shape of the dissipation signal becomes less broad. The full width at half maximum (a characteristic to describe pulse wave forms) of the dissipation signal decreases about 50% ± 14%. This shortening of the contraction-relaxation time is in good agreement with studies described above [37]. It is also interesting to note that changes in the dissipation signal were observed when the effect of 4 μM cytochalasin D on fibroblasts was studied by QCM-D in combination with light microscopy [21]. Upon addition of the cytomorphic agent, reversible (upon rinsing) changes in the dissipation were observed whilst the frequency stayed essentially constant. We assume that the observation that addition of cytochalasin D leads to changes in the dissipation, but not in frequency, is attributed to changes of the viscoelastic properties of the cells due to rupture of actin filaments. This assumption is supported by a recent study of Wu et al. showing by means of AFM that cytochalasin D leads to a 85% reduction of stiffness of cardiomyocytes [38]. We also note the correspondence with a previous study of the effects of cytoskeletal drugs on melanophores, where drug-induced changes in the actin layers were readily detected by QCM-D [25].

Taken together, our results suggest that acoustic sensing can be applied to single CMCs for surrogate measuring of the QT interval and other characteristics of the contractile machinery. These findings suggest that QCM-D has great potential as a tool for cardiotoxicological screening, where effects of compounds on the cardiomyocyte contractile machinery can be detected independently of whether the extracellular field potential is altered or not. In order to increase throughput and to reduce noise in the assay, the experimental set-up would need to be developed further, in particular with respect to liquid handling.

## Conclusion

We have demonstrated that QCM-D provides a versatile tool to study drug-induced effects on the contractile machinery, including chronotropic effects, prolongation and shortening of the QT-interval, as well as the effect of cytochalasin D on the contraction-relaxation cycles. Thus, effects of compounds on the cardiomyocyte contractile machinery can be detected independently of whether the extracellular field potential is altered or not. Our findings suggest great potential of QCM-D for cardiotoxicological screening.

## Supporting Information

S1 File Fig SI. QCM-D results obtained for individual clusters when exposed to E-4031. These data were underlying the corresponding values presented in Table 1. **Fig SII. QCM-D results obtained for individual clusters when exposed to nifedepine.** These data were underlying the corresponding values presented in Table 1. **Fig SIII. QCM-D results obtained for individual clusters when exposed to cytochalsin D.** These data were underlying values presented in the main text.
(DOCX)
