# Supplementary material for: Non-Invasive Acoustical sensing of Drug-Induced Effects on the Contractile Machinery of Human Cardiomyocyte Clusters
Source: PLoS One. 2015 May 11;10(5):e0125540. doi: 10.1371/journal.pone.0125540 (PMC4427273; doi:10.1371/journal.pone.0125540)
Supplement: S1 File — These data were underlying the corresponding values presented in Table 1. Fig SII. QCM-D results obtained for individual clusters when exposed to nifedepine. These data were underlying the corresponding values presented in Table 1. Fig SIII. QCM-D results obtained for individual clusters when exposed to cytochalsin D. These data were underlying values presented in the main text. (DOCX) [file pone.0125540.s001.docx]

**Supporting Information**

**Non-invasive acoustical sensing of drug-induced effects on the contractile machinery of human cardiomyocyte clusters**

Angelika Kunze,^1,#a^ Daniella Steel,^2,#b^ Kerstin Dahlenborg,^2^ Peter Sartipy,^2,3,#c^ and Sofia Svedhem^1,*^

^1^Department of Applied Physics, Chalmers University of Technology, Göteborg, Sweden

^2^Cellectis AB, Göteborg, Sweden

^3^Systems Biology Research Center, School of Bioscience, University of Skövde, Skövde, Sweden

**Statistical Evaluation**

Data presented in Table 1 and in the main text were obtained by monitoring of individual CMCs before and after addition of E-4031, nifedepine, or cytochalasin D. The following figures show the QCM-D time curves used for the analysis of the individual cluster. Note that the frequency and dissipation shifts are not presented as normalized values (as in the main text).

E-4031, Cluster 1


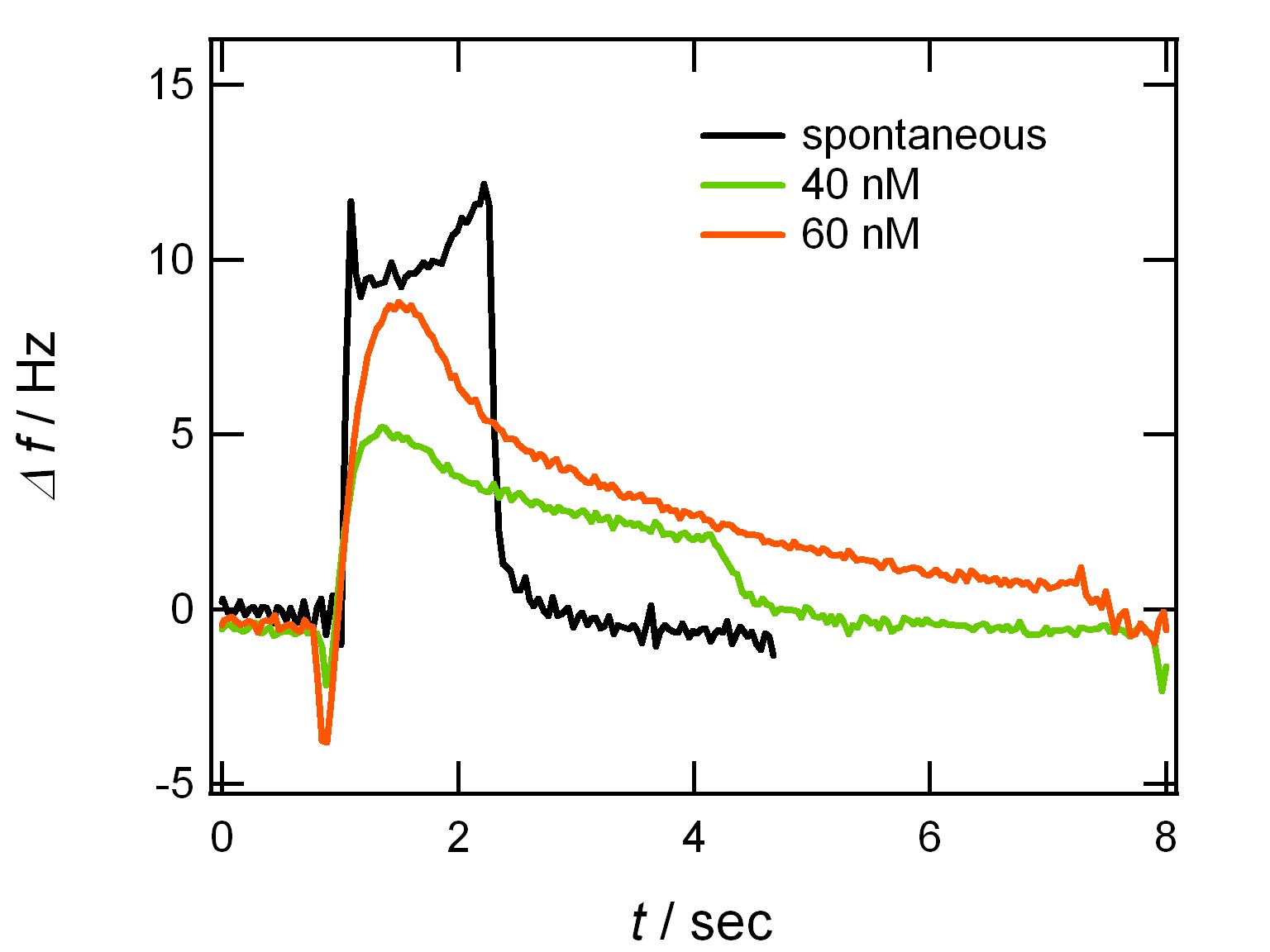

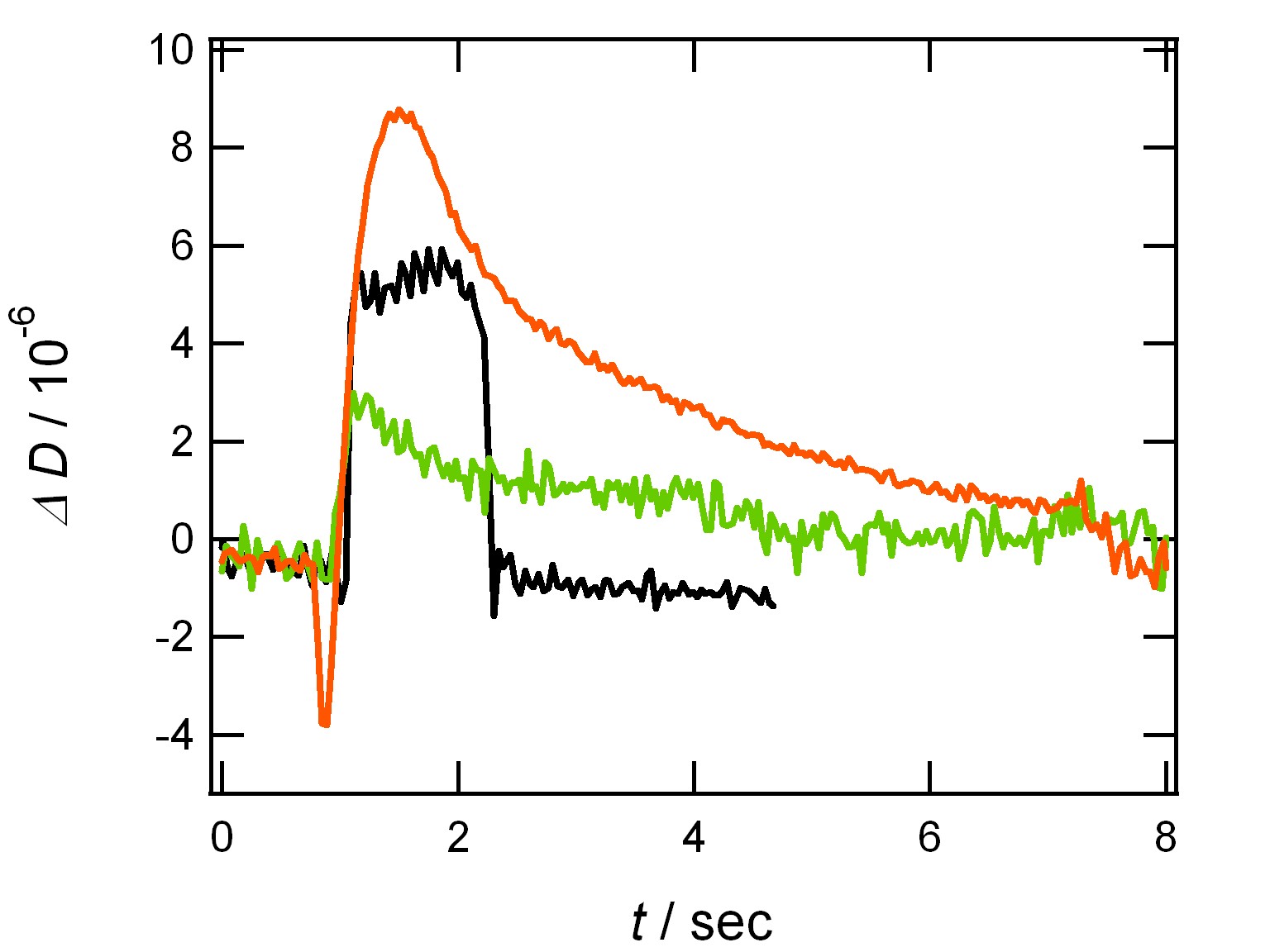


E-4031, Cluster 2


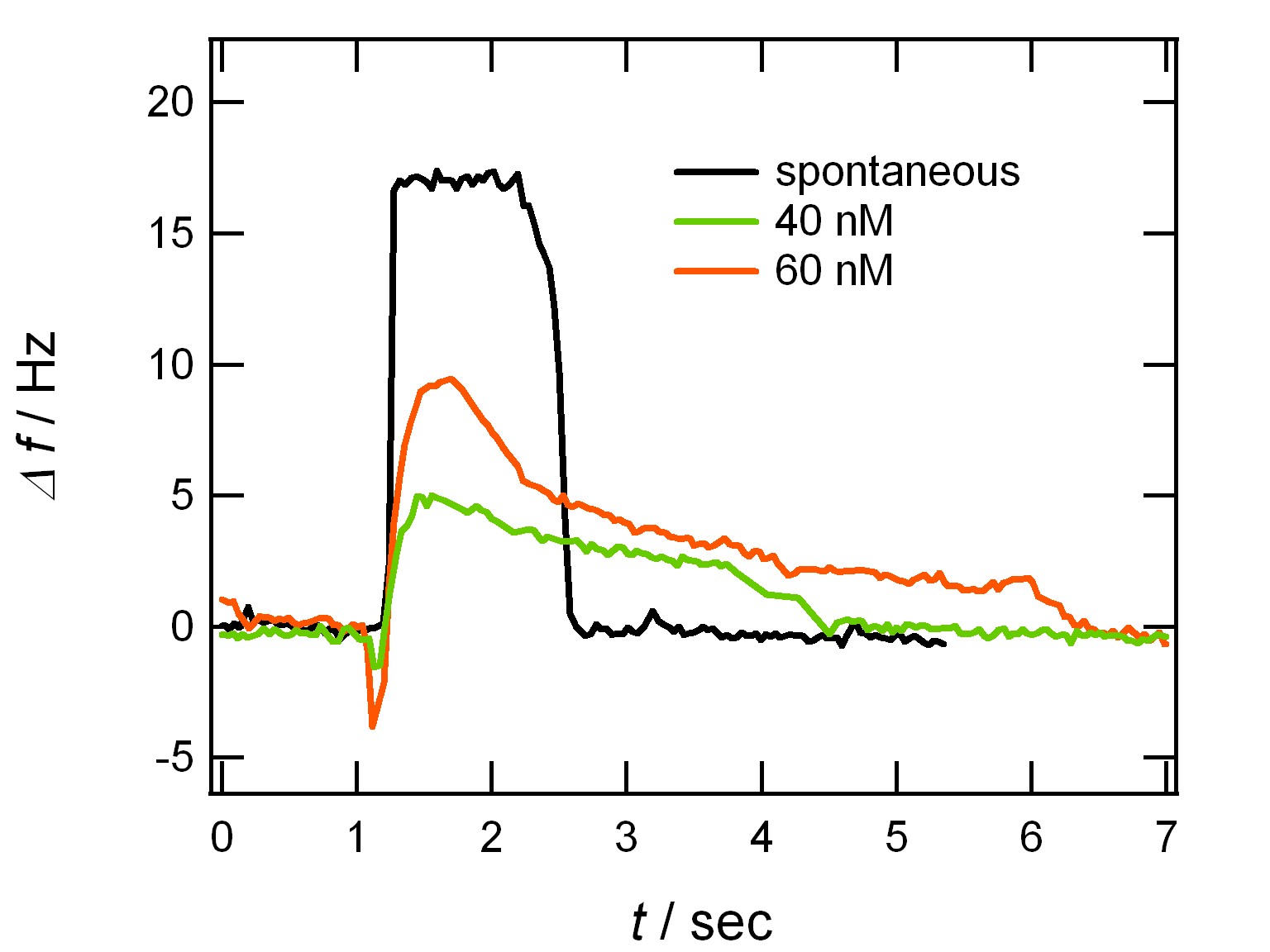

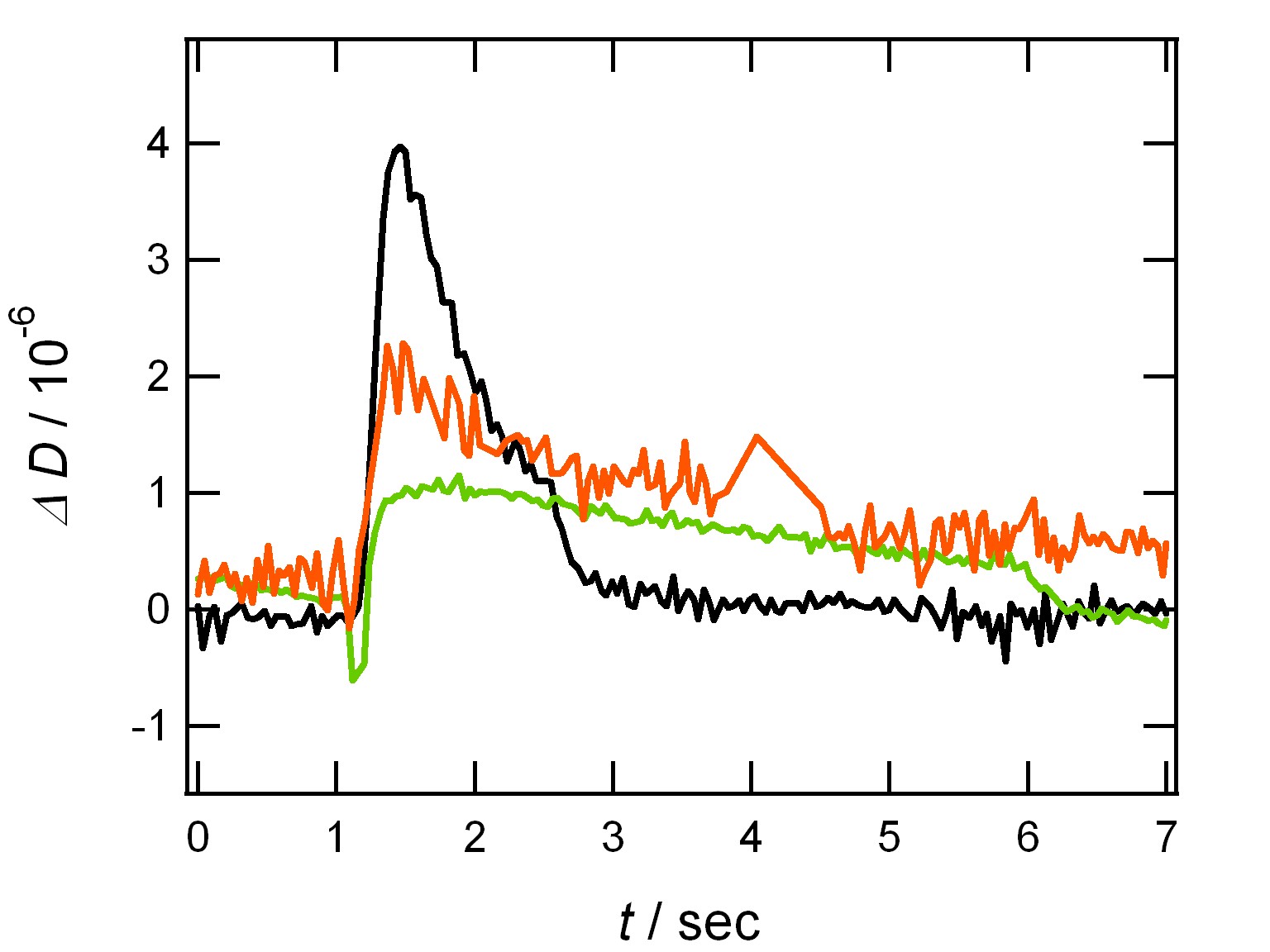


E-4031, Cluster 3


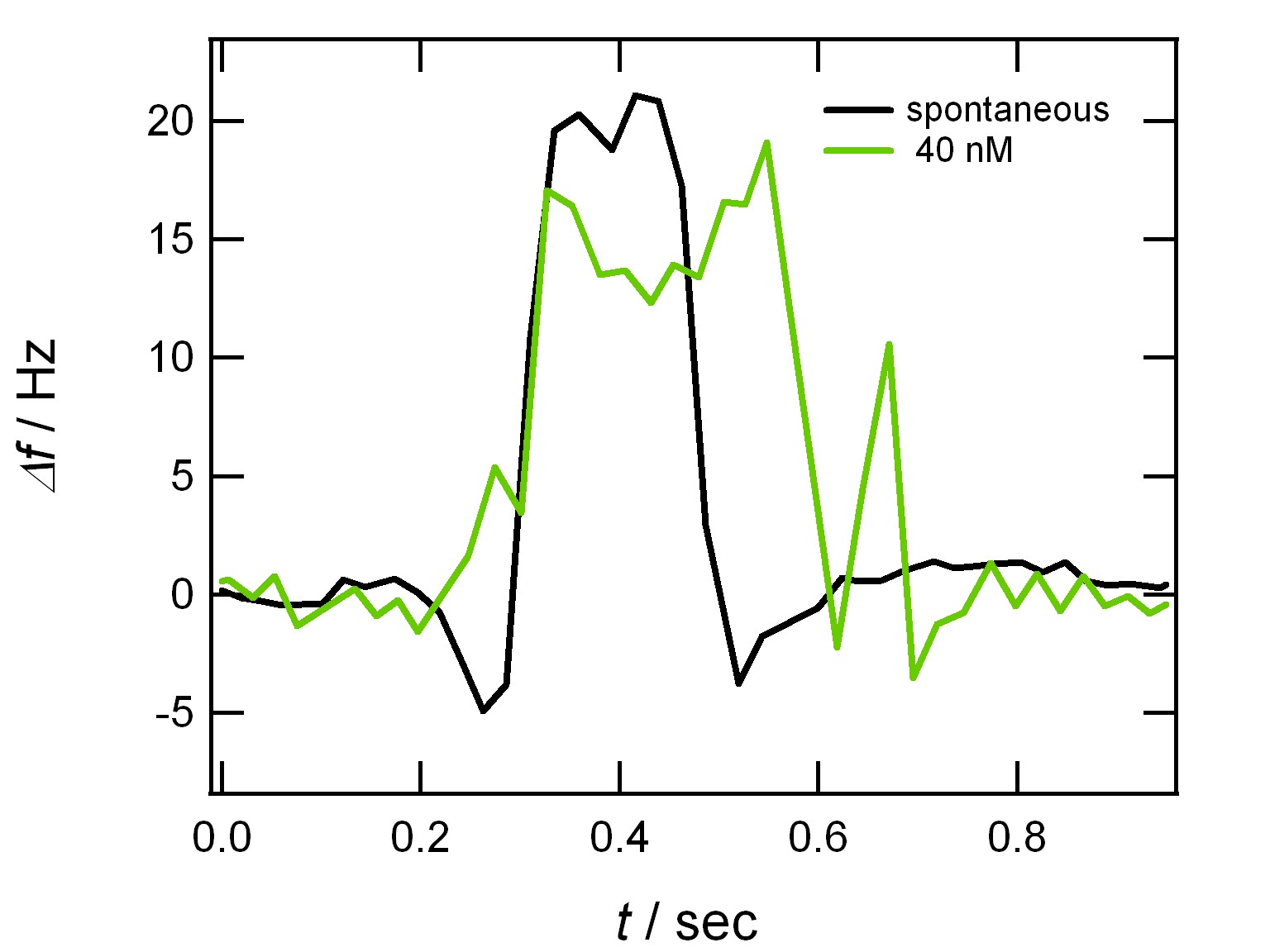

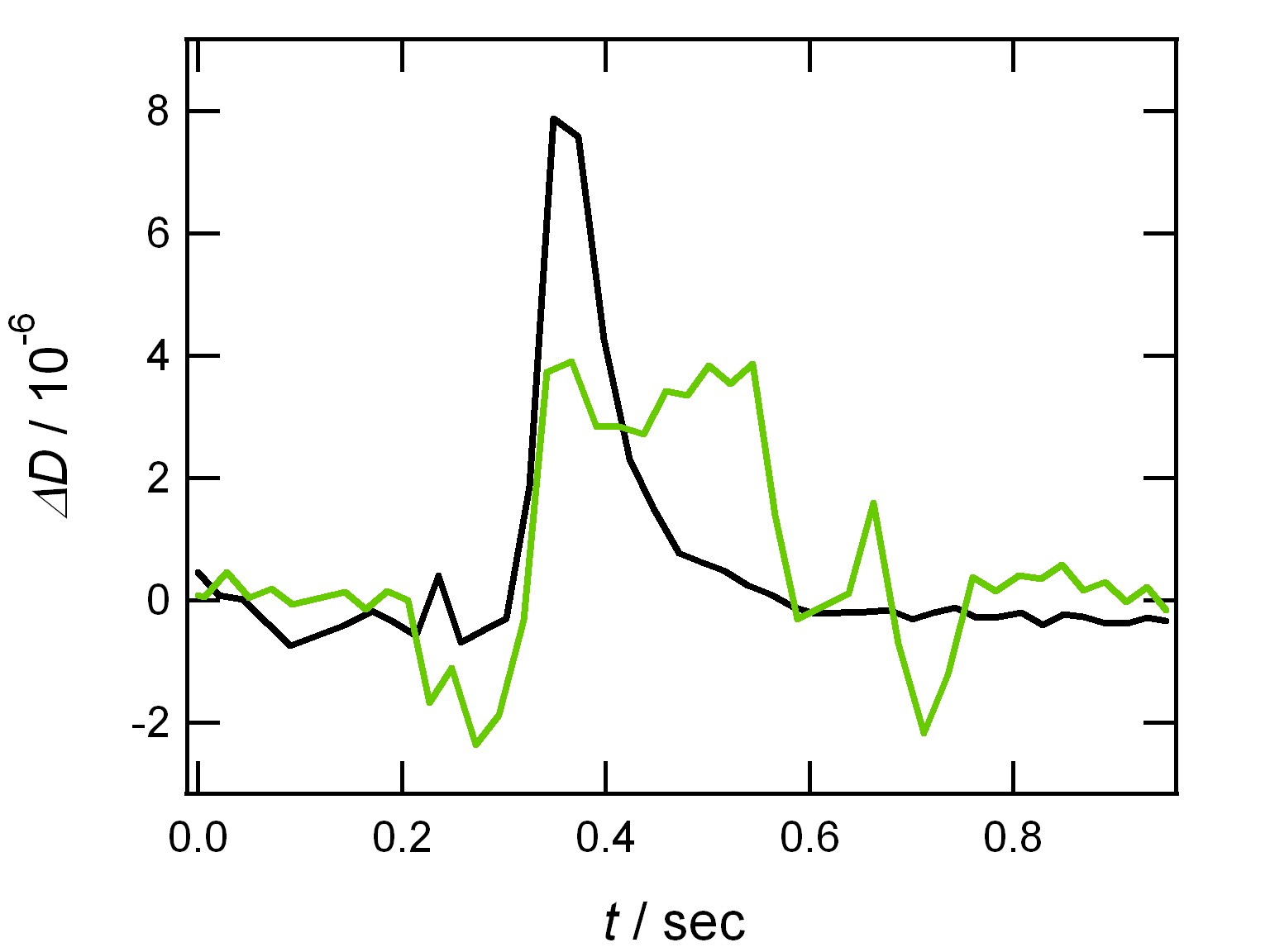


E-4031, Cluster 4


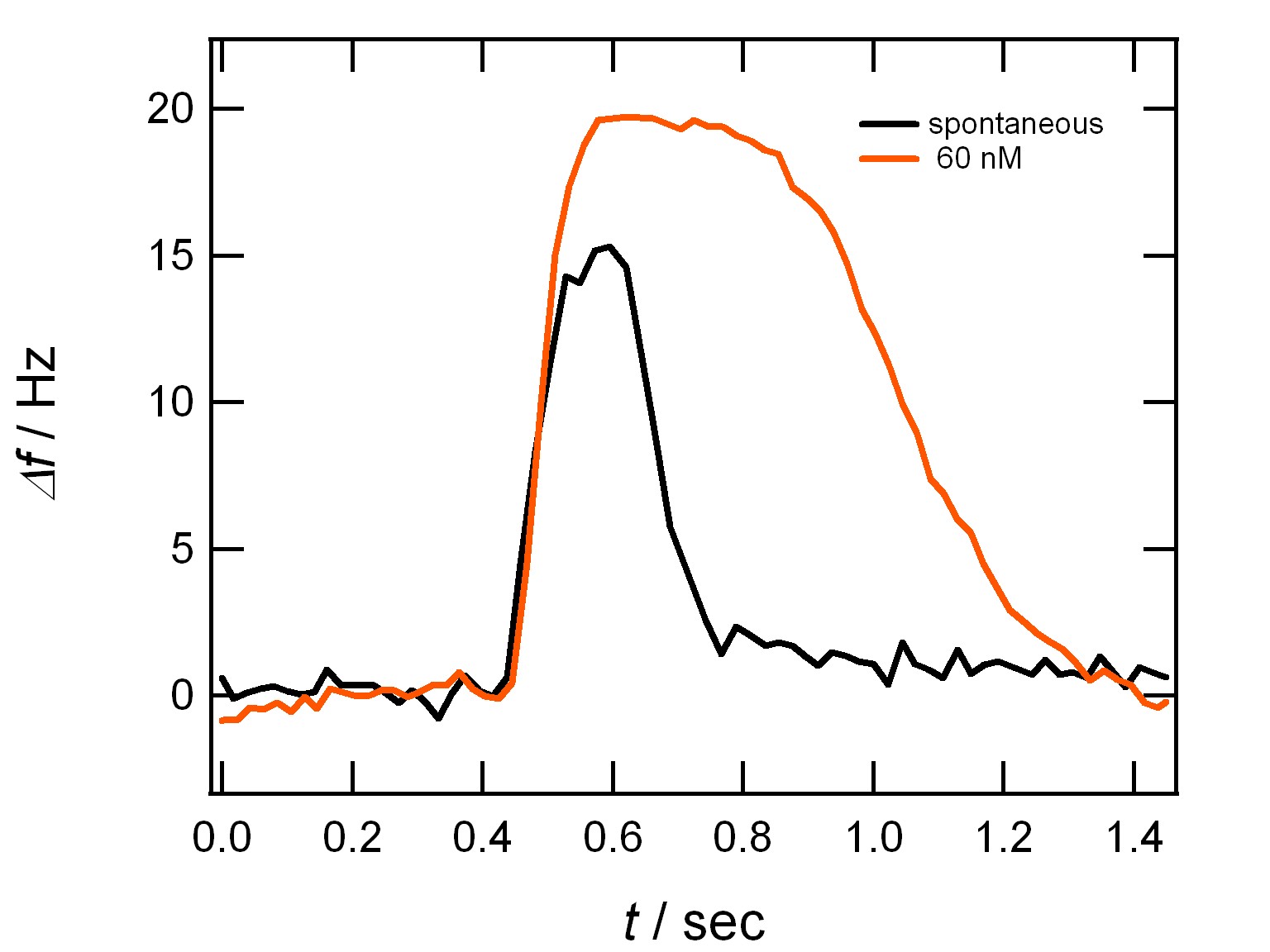

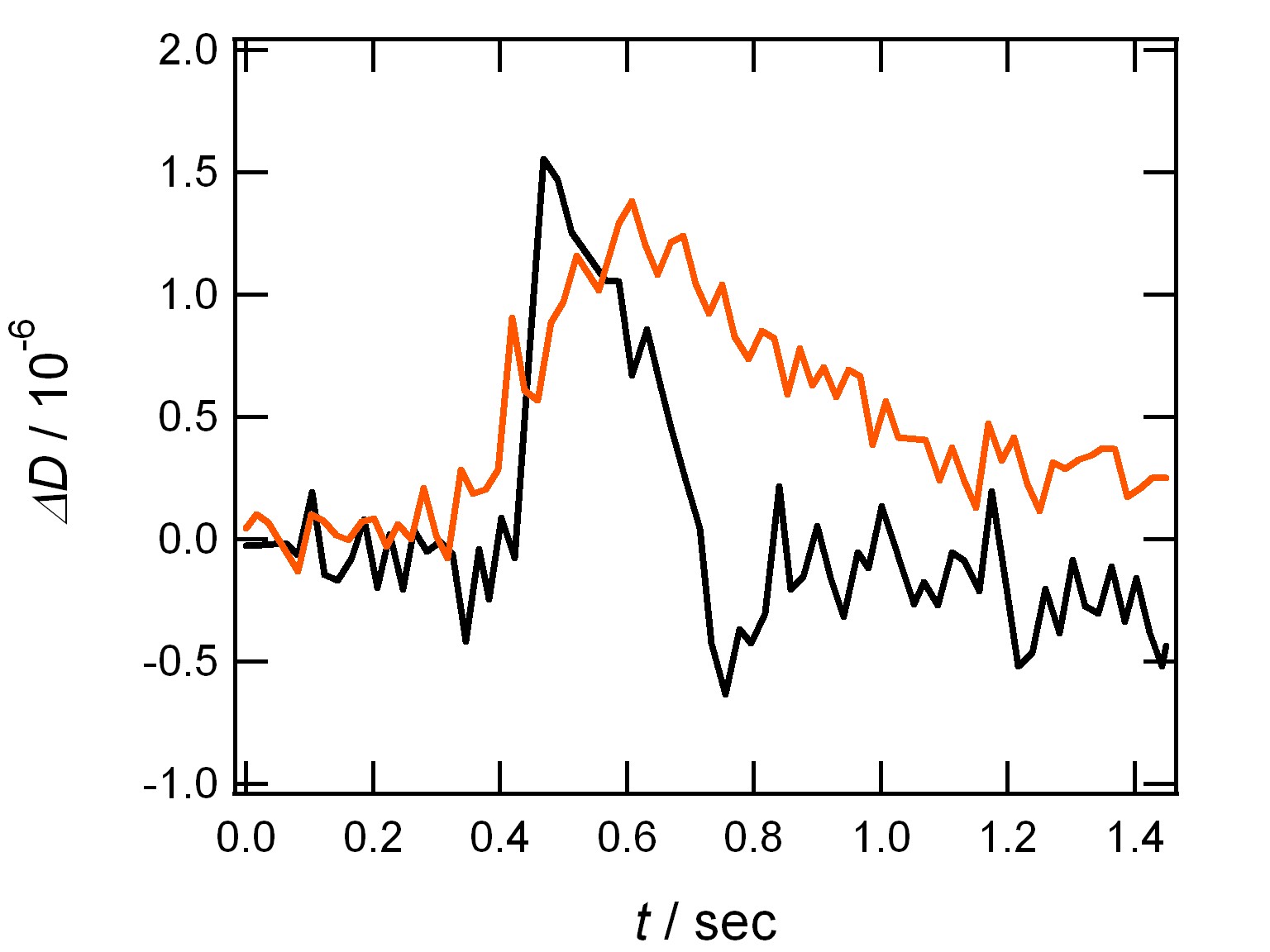


**Figure S1**. QCM-D results obtained for individual clusters when exposed to E-4031. These data were underlying the corresponding values presented in Table 1.

Nifedepine, Cluster1


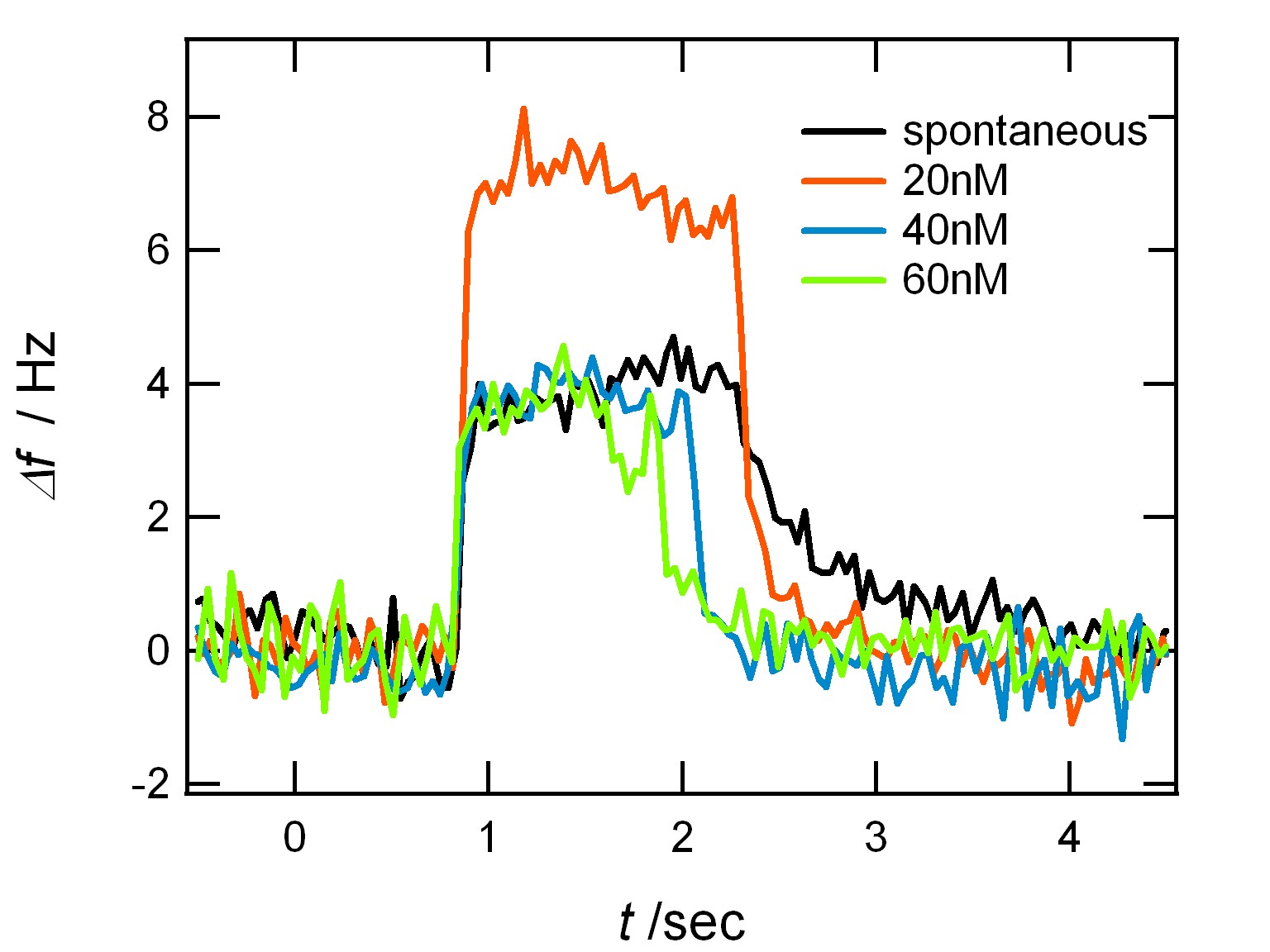

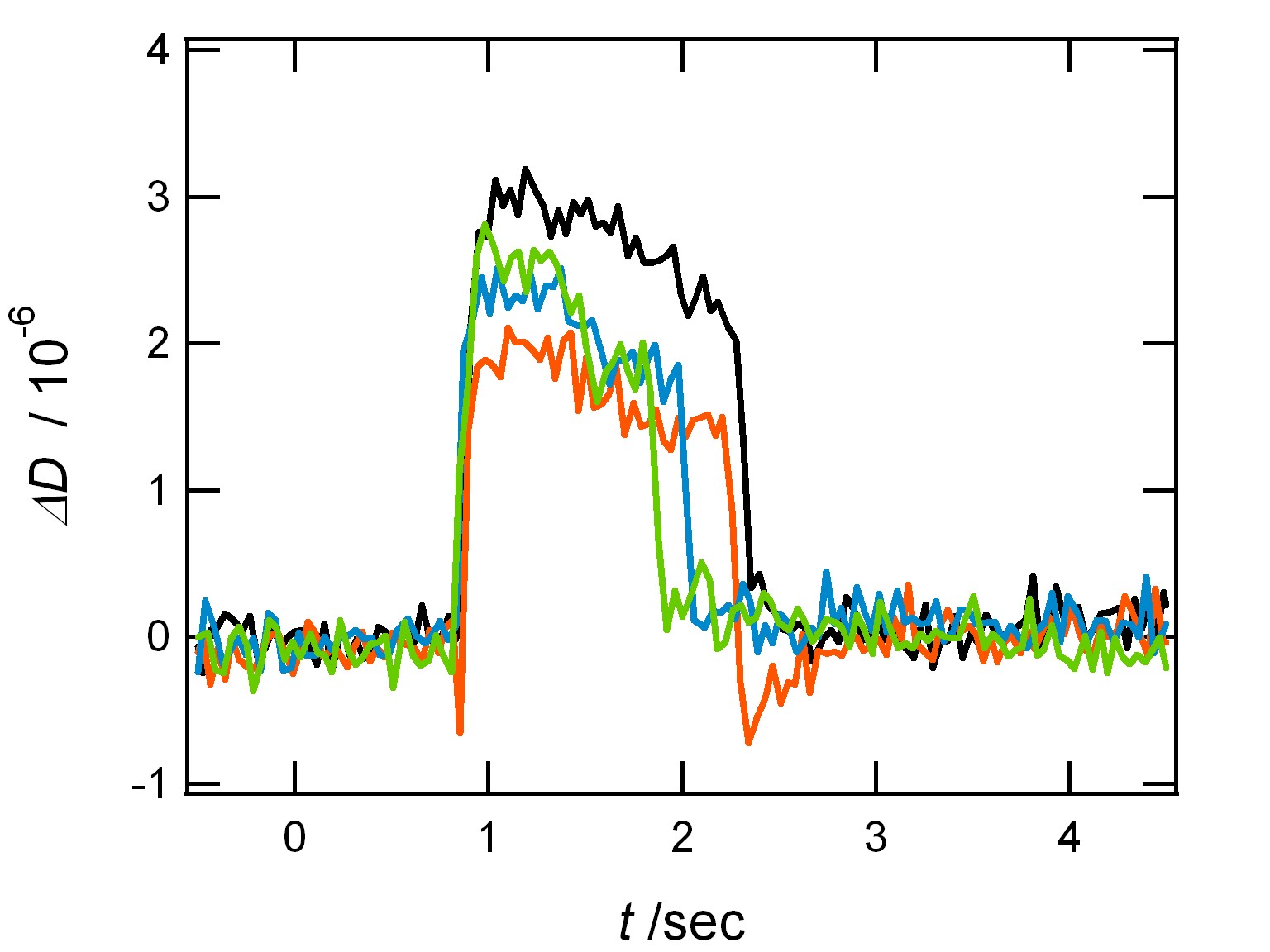


Nifedepine, Cluster 2


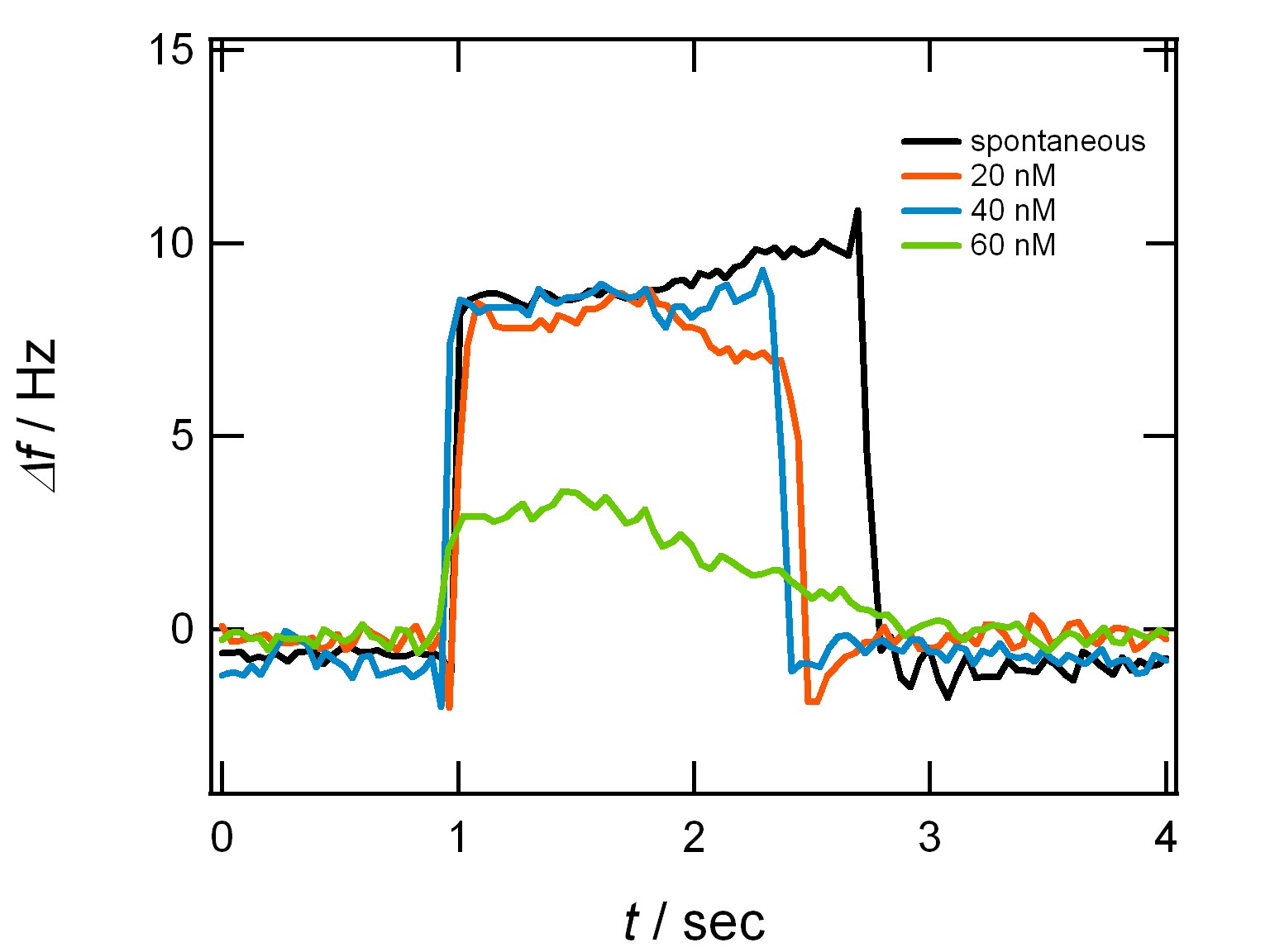

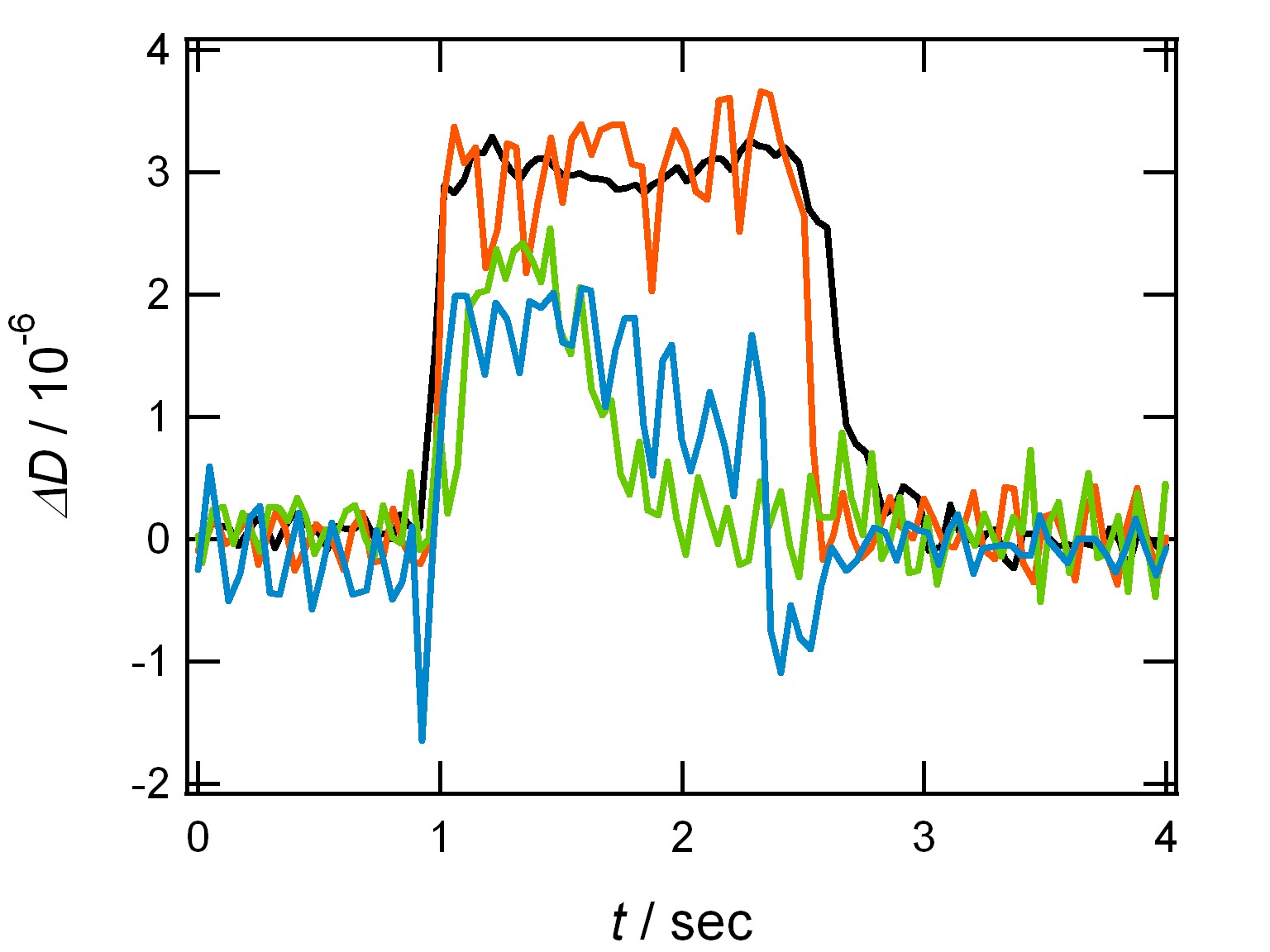


Nifedepine, Cluster 3


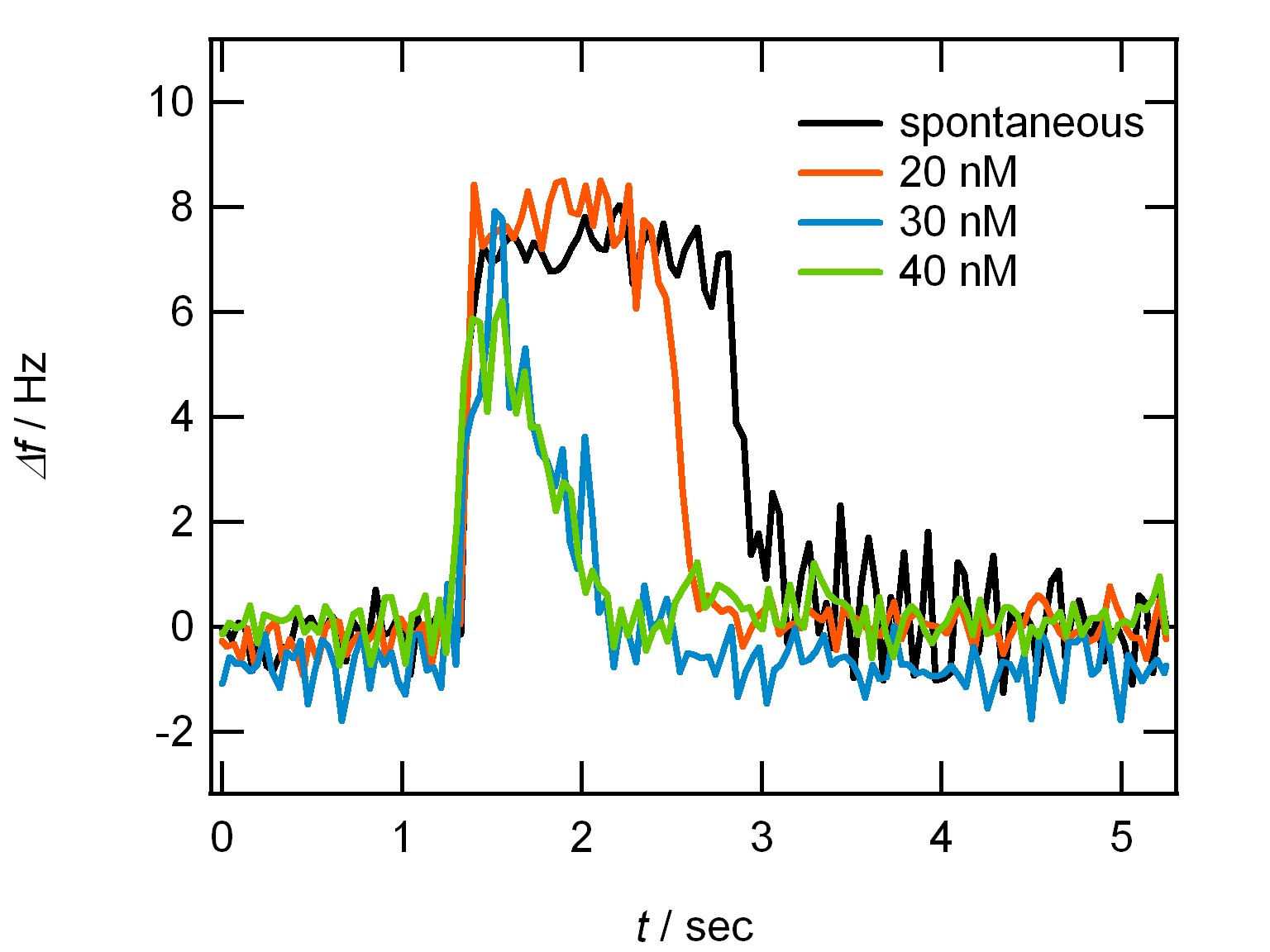

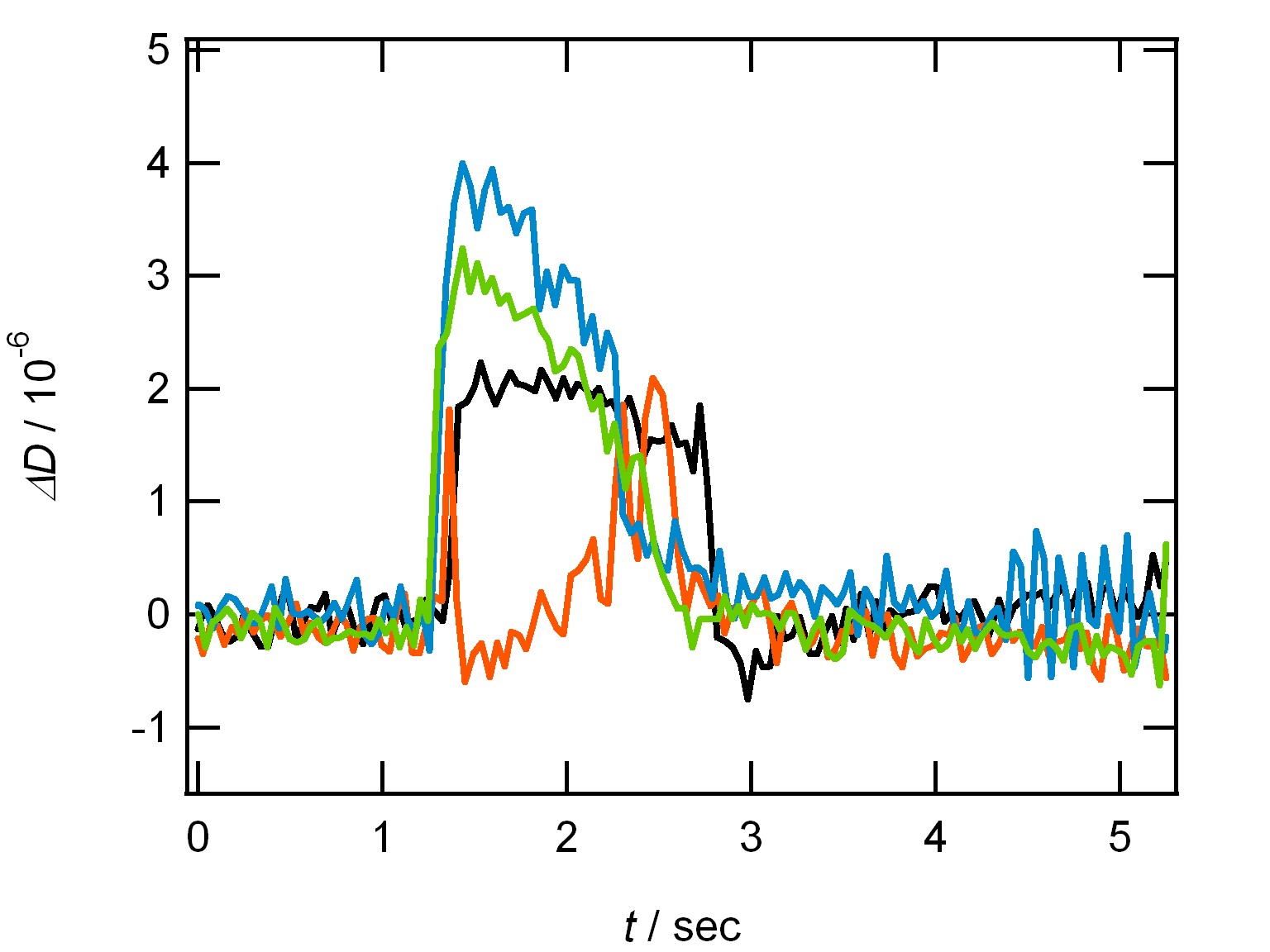


**Figure S2**. QCM-D results obtained for individual clusters when exposed to nifedepine. These data were underlying the corresponding values presented in Table 1.

Cytochalasin D, Cluster 1


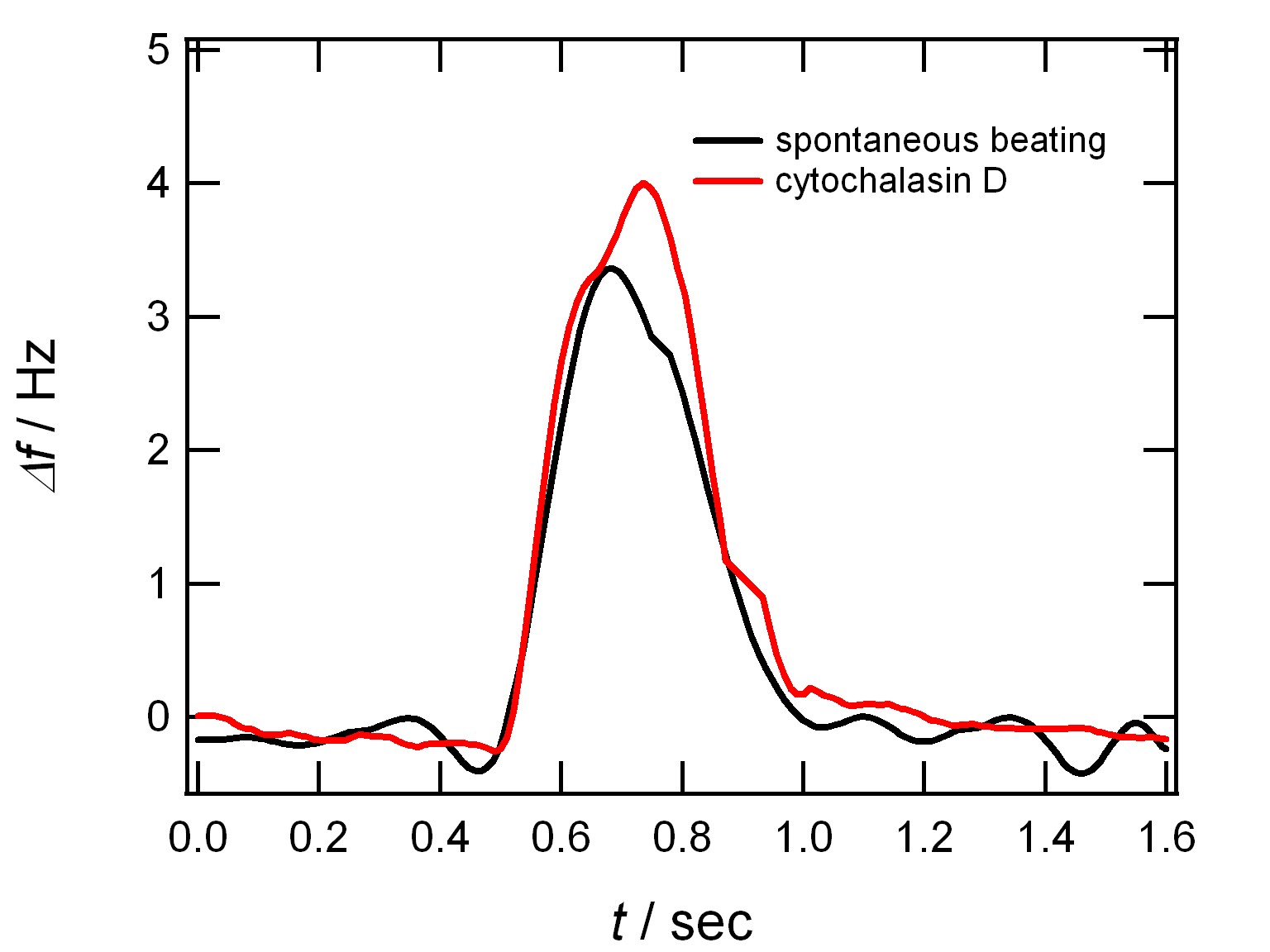

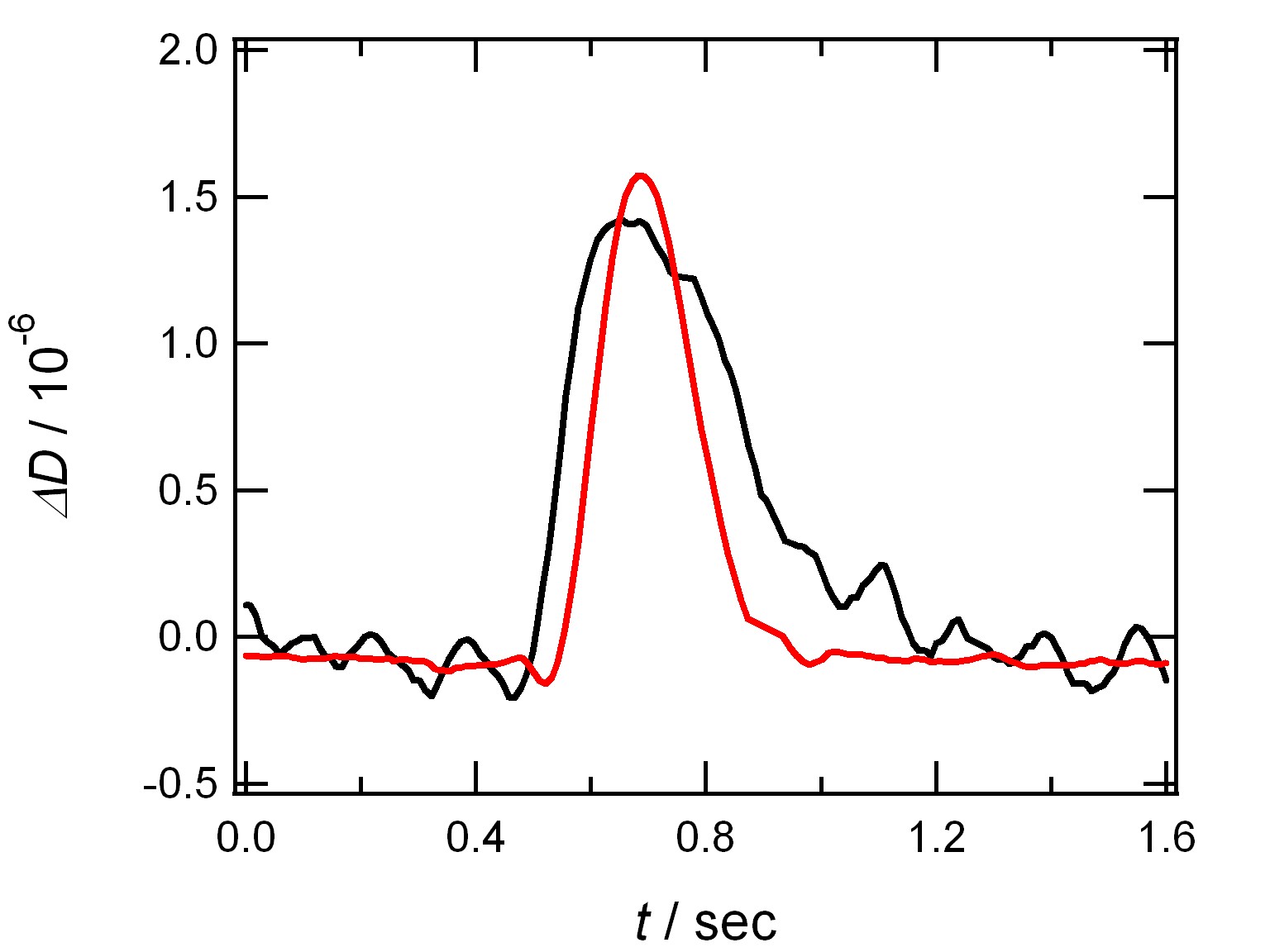


Cytochalasin D, Cluster 2


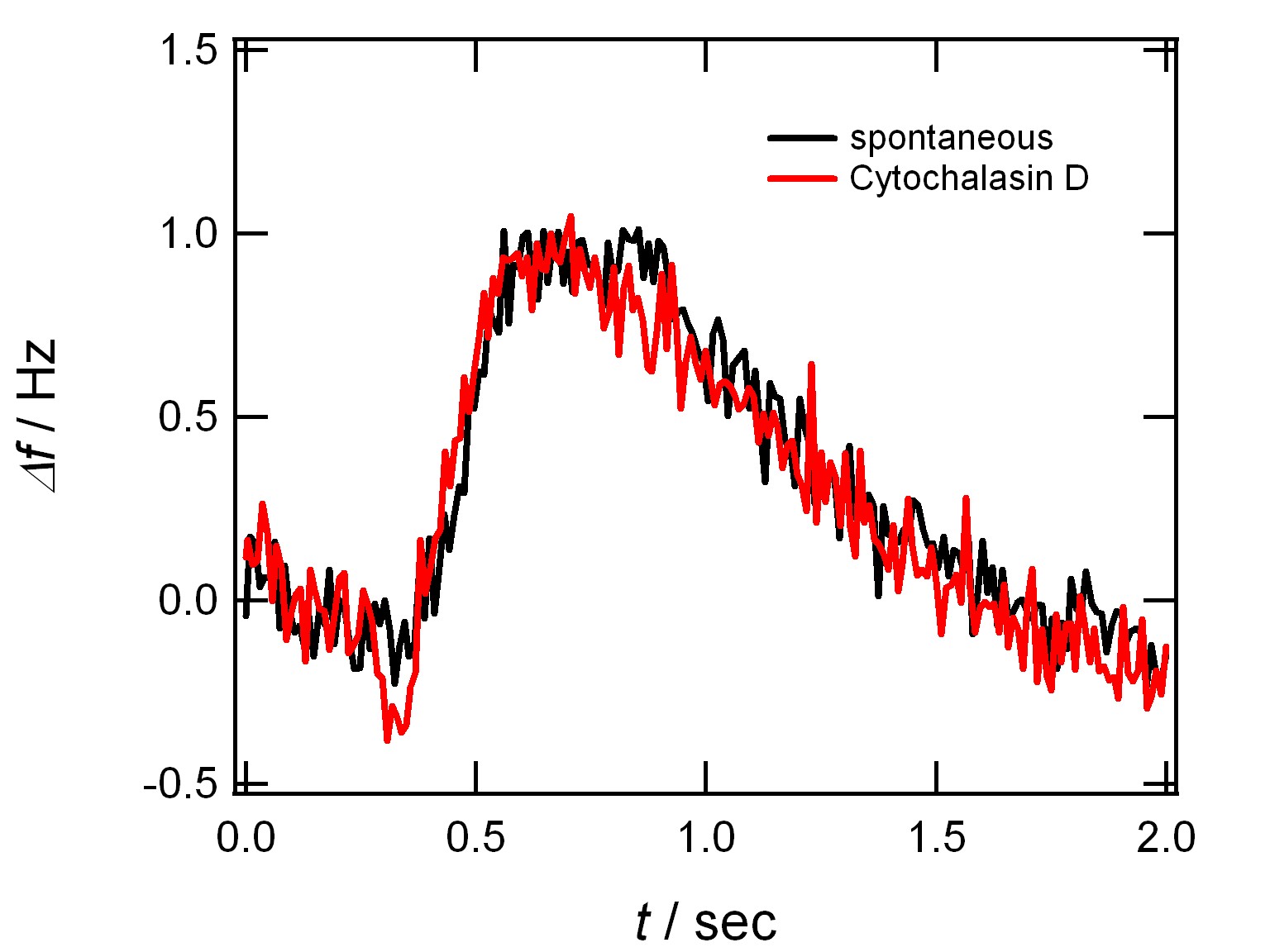

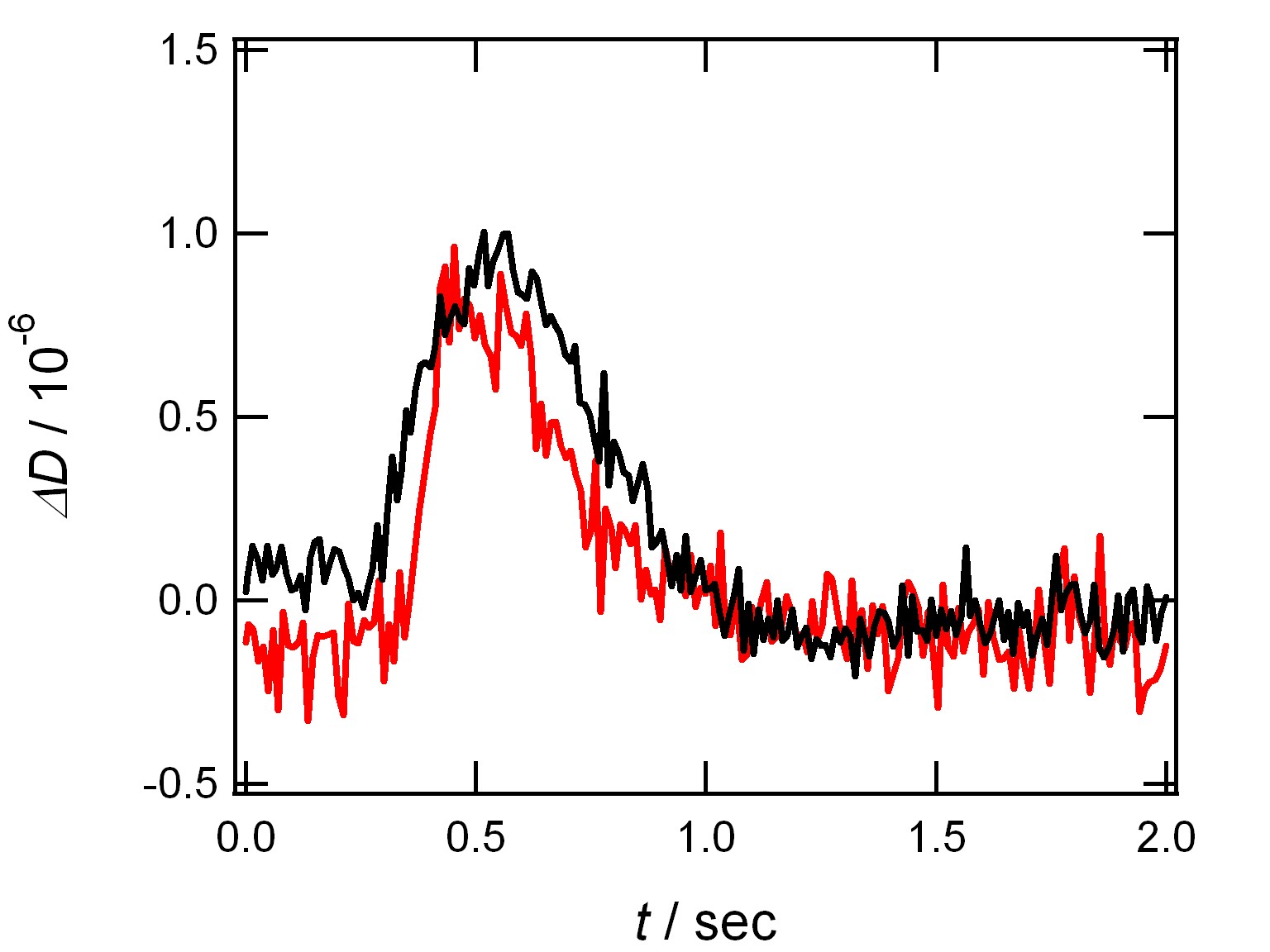


Cytochalasin D, Cluster 3


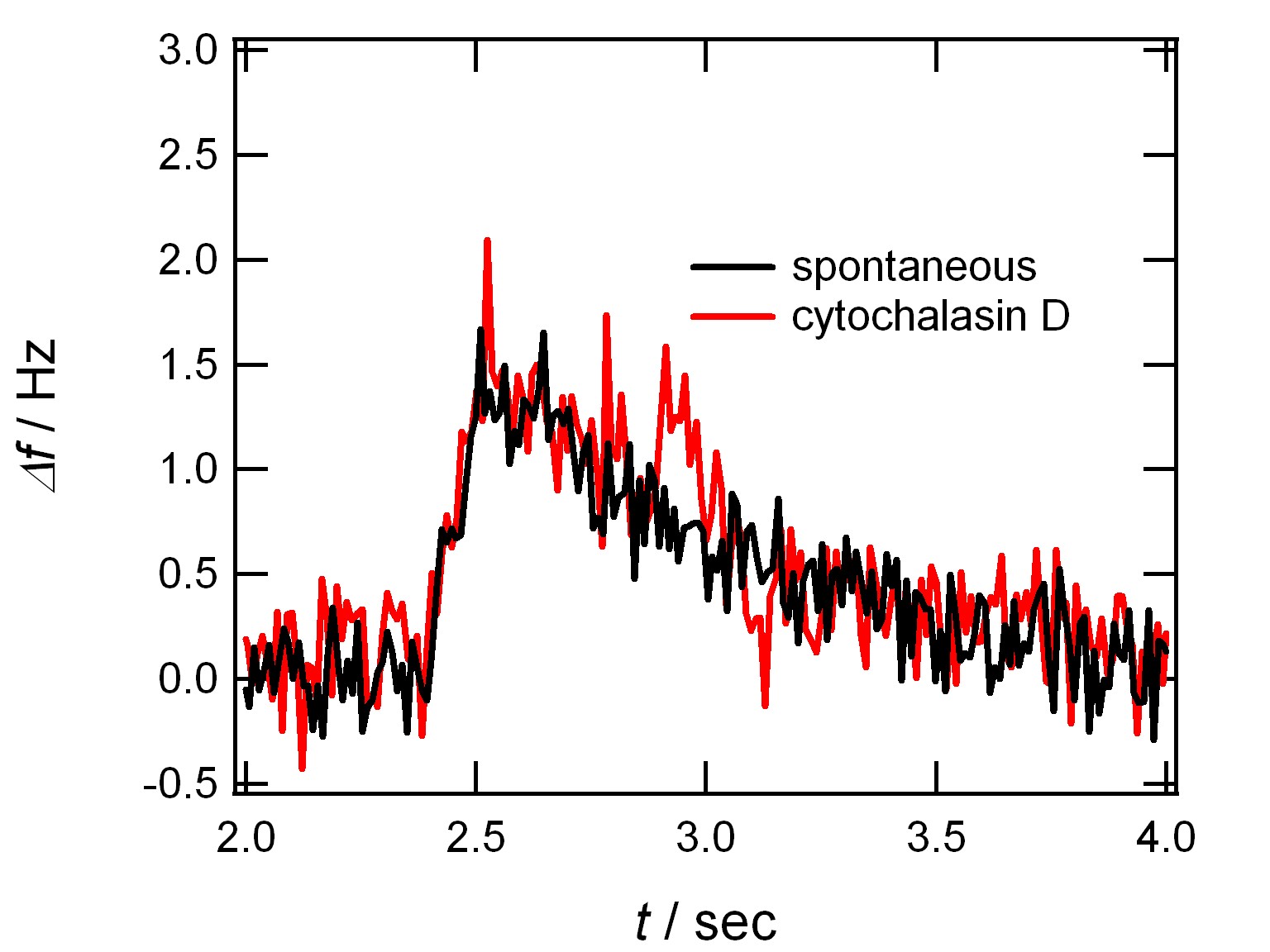

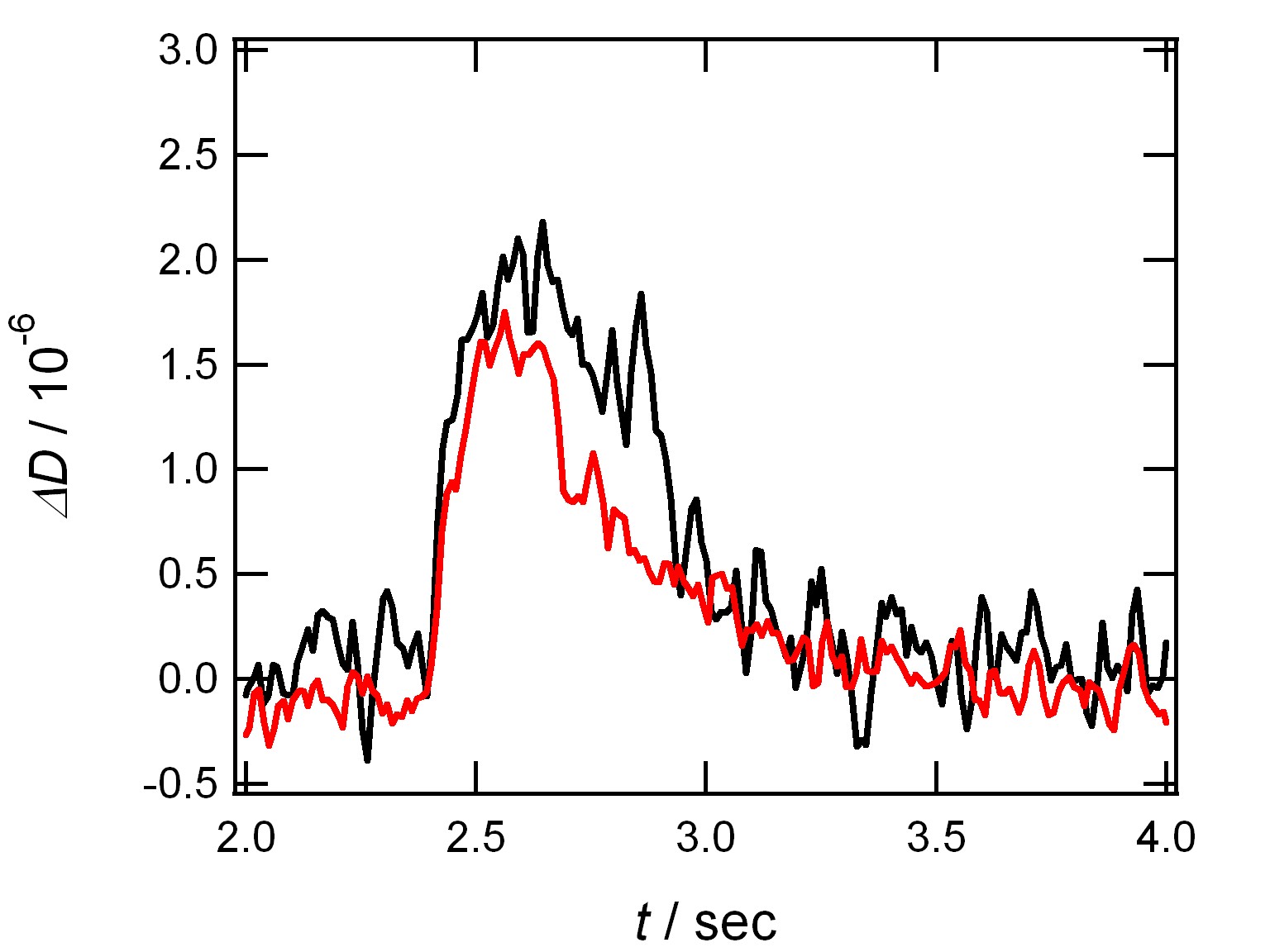


**Figure S3**. QCM-D results obtained for individual clusters when exposed to cytochalsin D. These data were underlying values presented in the main text.
